# Facteurs de risque cardiovasculaires au cours du lupus systémique

**DOI:** 10.11604/pamj.2015.22.367.7611

**Published:** 2015-12-14

**Authors:** Amel Harzallah, Mariem Hajji, Hayet Kaaroud, Fethi Ben Hamida, Taieb Ben Abdallah

**Affiliations:** 1 Hôpital Charles Nicolle, Médecine interne A, Tunis, Tunisie; 2 Laboratoire de Pathologie Rénale LR00SP01, Hôpital Charles Nicolle, Tunis, Tunisie

**Keywords:** Lupus systémique, risque cardiovasculaire, mortalité, anticorps antiphospholipides, dépistage, systemic lupus erythematosus, Cardiovascular risk, mortality, antiphospholipid antibodies, screening

## Abstract

Cette étude a pour objectif d’évaluer la fréquence des facteurs de risque cardiovasculaires au cours du lupus et de préciser leur prévalence. Etude rétrospective portant sur 250 patients ayant un lupus, diagnostiqué selon les critères de l'ACR, hospitalisés entre 1970 et 2013. Les données cliniques et para cliniques ont été recueillies à partir des observations médicales. Il s'agit de 228 femmes et 22 hommes d’âge moyen au diagnostic du lupus de 30, 32 ans (extrêmes: 16-69). La durée moyenne du suivi des patients était de 64 mois (extrêmes: 7 jours- 382mois). Quatre vingt dix patients (36%) étaient hypertendus, 74% avaient une hypercholestérolémie et 22% étaient diabétiques. Pour les autres facteurs de risque cardiovasculaire traditionnels, un âge > 50 ans a été retrouvé dans 40% des cas, le sexe masculin dans 8% des cas, l'obésité dans 76% des cas et le tabagisme dans 11% des cas. Les facteurs de risque surajoutés sont représentés par la présence des anticorps antiphospholipides (47% des cas), la néphropathie lupique (49% des cas), l'insuffisance rénale (42% des cas), la corticothérapie au long cours (74% des cas) et la chronicité de la maladie dans 35% des cas. Les complications cardiovasculaires retrouvées dans notre série étaient: les accidents vasculaires cérébraux (2%) et l'insuffisance coronarienne (5,6%). Devant l'importance du risque cardiovasculaire au cours du lupus, une surveillance rapprochée des facteurs de risque cardio-vasculaires semble primordiale chez les lupiques.

## Introduction

La mortalité cardiovasculaire est la première cause de mortalité au cours du lupus érythémateux systémique (LES), soit 50 fois plus que la population générale. Elle est estimée entre 10 et 20% de la mortalité globale au cours du lupus [1–3]. Le risque d′évènements cardio-vasculaires est 2 à 3 fois plus élevé au cours de cette maladie associé à une mortalité coronarienne et neuro-vasculaire augmentées par rapport à des populations appariées pour l′âge et le sexe [1, 2]. Ainsi, plusieurs études ont montré que le risque d′infarctus est multiplié par 2 à 10 au cours du lupus comparativement à des témoins appariés et que le risque d′accident vasculaire cérébral est multiplié par 7,9 [4, 5]. Les facteurs incriminés sont multiples. On retrouve une plus forte prévalence des facteurs de risque cardio-vasculaires (FDRCV) traditionnels que dans la population générale mais aussi des facteurs propres à la maladie. L′objectif de notre étude était d′identifier les différents facteurs de risque cardiovasculaire dans une cohorte de patients suivis pour un LES et de préciser leur prévalence.

## Méthodes

Il s'agit d'une étude rétrospective portant sur les patients âgés de plus de 18 ans, ayant un LES, qui ont été hospitalisés dans notre service entre 1970 et 2013. Le diagnostic de lupus a été fait selon les critères de L'American College of Rheumatology (ACR) révisés en 1997 [6]. Les données démographiques, cliniques et para cliniques ont été recueillies à partir des observations médicales. La durée de suivi des patients a été définie par la période séparant le moment où le diagnostic de lupus a été posé et la date des dernières nouvelles. La fonction rénale a été estimée par la formule MDRD. L’âge est exprimé en années et la créatinine sérique (Cr) en µmol/l. L'obésité a été définie selon les critères de l'organisation mondiale de la santé (OMS) [7]. La biopsie rénale a été réalisée dans notre service et évaluée selon la classification de la néphropathie lupique de la société internationale de néphrologie et de pathologie rénale (ISN/RPS) [8].

### Analyse statistique

Les résultats ont été exprimés soit en termes de nombre de cas et/ou de pourcentage pour les variables qualitatives et en termes de moyennes pour les variables quantitatives La collecte des données, l'analyse descriptive et analytique ont été réalisées à l'aide du logiciel SPSS 11. Nous avons utilisé le test de Chi2 pour la comparaison des pourcentages et le test d'analyse des variances ANNOVA pour la comparaison des moyennes. Une différence est déclarée significative à chaque fois que la valeur de p est inférieure à 0,05.

## Résultats

On a inclus dans notre étude 250 patients. Il s'agit de 228 femmes et 22 hommes d’âge moyen au diagnostic du lupus de 30,32 ans (extrêmes: 16-69 ans). La durée moyenne du suivi des patients était de 64 mois (extrêmes: 7 jours-382 mois). Le taux de survie rénale à 10 ans était de 82,7%. La survie globale des patients à 20 ans était de 94,6%. Les principales caractéristiques cliniques et biologiques de nos patients sont résumées dans le Tableau 1. Au moment du diagnostic, on a noté une HTA dans 36% des cas, un diabète dans 22% des cas et une obésité dans 76% des cas avec un indice de masse corporelle moyen de 26,4 Kg/m^2^(extrêmes: 18,9-32,7). Un tabagisme a été rapporté dans 11% des cas. Les FDRCV traditionnels retrouvés dans notre étude sont résumés dans le Tableau 2. Une néphropathie glomérulaire a été objectivée dans 49% des cas avec une protéinurie moyenne à 5,2 g/24h (extrêmes: 2,8-7,4). Une insuffisance rénale a été notée dans 42% des cas avec une créatininémie moyenne à 451 µmol/l. Le taux de cholestérol moyen était de 3,42 g/L (extrêmes: 1,6-6,2) avec une hypercholestérolémie dans 74% des cas. Une corticothérapie orale a été prescrite dans 74% des cas associée à un traitement immunosuppresseur à base de cyclophosphamide dans 19,6% des cas et de mycophénolate mofétil dans 12,8% des cas. Au cours du suivi, on a retrouvé une HTA dans 40,4% des cas, un diabète dans 34,8% des cas et une dyslipidémie dans 24% des cas. Après une analyse univariée, les FDRCV surajoutés identifiés dans notre étude étaient le syndrome des antiphospholipides (47%) (p=0,01), la néphropathie lupique (49%) (p=0,02), l'insuffisance rénale (42%) (p=0,018), la corticothérapie au long cours (74%) (p=0,009) et la chronicité de la maladie (évolution du lupus >10 ans) dans 35% des cas (p=0,014). Les complications cardiovasculaires retrouvées dans notre série ont été principalement les accidents vasculaires cérébraux (2%) et l'insuffisance coronarienne (5,6%). On a retrouvé que les évènements cardio-vasculaires étaient plus fréquents chez les lupiques ayant des facteurs de risque cardio-vasculaire: infarctus du myocarde (3,4% vs 1,9%), angor (2,7% vs 1,3%), accident vasculaire cérébral (1,7% vs 0,3%), accident ischémique transitoire (1,2% vs 0,8%). La survie chez les patients n'ayant pas des facteurs de risque cardio-vasculaire était meilleure (97% vs 88%).

**Tableau 1:** Principales caractéristiques cliniques et biologiques de nos malades

Paramètres	Nombre	Pourcentage (%)
Sexe masculin	22	8,8
Atteinte cutanée	213	85,2
Néphropathie glomérulaire	196	78,4
Pleurésie	97	38,8
Péricardite	89	35,6
Atteinte neurologique	47	18,8
Atteinte psychiatrique	39	15,6
Thrombose vasculaire	114	45,6
Anémie	245	98
Lymphopénie	189	75,6
Thrombopénie	134	53,6
AAN	250	100
Ac antiDNA natifs	238	95,2
Ac anticardiolipines	116	46,4
Ac anticoagulant circulant	105	42
Baisse du CH50	171	68,4
Cryoglobulinémie	41	16,4
Syndrome néphrotique	83	33,2
Insuffisance rénale	105	42
Corticothérapie	185	74

**Tableau 2:** Facteurs de risque cardio-vasculaires traditionnels de nos patients

Facteur de risque	Nombre	Pourcentage (%)
Age >50 ans	100	40
Tabagisme	28	11
HTA	90	56
Diabète	55	22
Obésité	190	76
hypercholestérolémie	185	74

## Discussion

Le pronostic du lupus s'est actuellement considérablement amélioré du fait d'un diagnostic plus précoce, d'une amélioration de la prise en charge des complications infectieuses et du développement de l'arsenal thérapeutique. Toutefois, la survenue d′une athérosclérose précoce au cours du lupus est actuellement établie. Cette athérosclérose est d'autant plus inhabituelle qu'elle se voit chez des femmes jeunes le plus souvent sans facteur de risque cardiovasculaire identifié [9]. Le caractère bimodal de la mortalité au cours du lupus a ainsi été décrit dés 1976 avec une mortalité précoce en rapport avec la maladie et une mortalité tardive cardiovasculaire [3]. Ce risque accru de survenue de complications cardio-vasculaires chez les patients lupiques a deux causes principales qui sont le développement d'une maladie athéromateuse accélérée d'une part et la tendance prothrombotique d'autre part. Deux groupes de facteurs sont ainsi incriminés: les facteurs de risque classiques (ceux de l′étude de Framingham) et des facteurs propres à la maladie. En effet, Les facteurs de risque classiques de l'athérosclérose établis par l’étude de Framingham dans la population générale sont significativement plus fréquents chez les lupiques [10]. Les premiers résultats de notre étude montrent effectivement que les facteurs de risque cardiovasculaires sont largement présents et que le risque d′événement coronarien à 10 ans selon le score de Framingham est > 5% chez 1/3 de nos patients. Ainsi, plusieurs études ont retrouvé chez les lupiques une plus forte prévalence de ces facteurs de risque cardiovasculaires traditionnels comme l'HTA, la dyslipidémie, l'intoxication tabagique et l'insuffisance rénale, que dans les populations témoins [11]. L'HTA fréquente chez les patients lupiques est un facteur de risque indépendant d'athérosclérose et d’évènements cardio-vasculaires [12]. Dans notre série une HTA a été retrouvée dans 36% des cas et un diabète dans 22% des cas. Dans certaines études, le diabète chez les lupiques a été aussi corrélé aux complications cardio-vasculaires [9]. En effet, au cours du lupus, l'insulinémie à jeun est plus élevée par rapport à la population générale. Il existe également une sensibilité diminuée à l'insuline et une baisse de la fonction sécrétoire des cellules béta pancréatiques [13]. Le syndrome métabolique qui est également un marqueur important de morbi-mortalité cardio-vasculaire est 2 à 3 fois plus fréquent chez les lupiques surtout de moins de 40 ans [14]. Parmi les autres FDRCV, une obésité a été retrouvée dans 76% des cas dans notre étude. Elle est indépendamment associée au risque cardio-vasculaire au cours du lupus [15]. Ainsi, un index de masse corporelle élevé est corrélé à la présence de plaques d'athérome carotidiennes [16].

En plus de ces facteurs de risque habituels, il existe également des facteurs de risque inhérents à la maladie lupique qui ont été identifiés dans la littérature. En effet, le lupus est une maladie inflammatoire chronique qui s'accompagne d'une hyperproduction de cytokines pro-inflammatoires telles que le TNF alpha et l'interleukine 6 dont les taux élevés sont corrélés à la présence de calcifications coronaires [17]. Les auto-anticorps au cours du lupus peuvent aussi modifier le profil lipidique, provoquer la synthèse de facteur tissulaire, favoriser la coagulation et induire une apoptose endothéliale. L'ancienneté de la maladie lupique semble également être un facteur de risque important d'une athérosclérose prématurée. Ainsi, un lupus évoluant depuis plus de 10 ans s'accompagne d'un athérome carotidien significativement plus important et plus évolutif [18]. Dans notre étude, une évolution du lupus depuis plus de 10 ans a été retrouvée dans 35% des cas. Plusieurs indices d’évaluation de l'activité de la maladie lupique comme le SLICC apparaissent aussi comme des facteurs prédictifs indépendants d’évènements cardio-vasculaires [16]. L'existence d'une néphropathie lupique semble jouer un rôle dans la genèse de l'athérosclérose au cours du lupus. En effet, plusieurs études ont corrélé la survenue d’évènements cardio-vasculaires et la présence ainsi que la progression des plaques carotidiennes à l'existence d'une protéinurie [19]. Dans notre série une néphropathie glomérulaire était présente dans 49% des cas. Les traitements de la maladie lupique influencent aussi largement les facteurs de risque traditionnels, puisque les corticoïdes sont reconnus pour induire ou majorer une HTA, mais également pour favoriser le développement d'une obésité, d'un diabète ou d'une dyslipidémie. En effet, bien que le traitement corticoïde a largement contribué à l'amélioration de la prise en charge des patients lupiques, la durée de la corticothérapie a été retrouvée significativement plus longue quand on compare les malades avec et sans évènements cardiovasculaires. Ainsi, une durée d'exposition à des doses > 20 mg/l est plus à risque d’évènements cardio-vasculaires [10]. Concernant les autres thérapeutiques au cours du lupus, l'utilisation du cyclophosphamide s'accompagne de moins de plaques carotidiennes et d'une progression moindre de ces plaques alors que le mycophénolate mofétil semble avoir un effet athéro-protecteur chez des souris modèle d'athérosclérose et lupus [19]. Les antipaludéens de synthèse qui sont actuellement la pierre angulaire du traitement de fond du lupus semblent avoir également un rôle athéro-protecteur qui a été rapporté par plusieurs études [20]. En effet, ils permettent de diminuer l'activité de la maladie et les atteintes organiques irréversibles et d'améliorer la mortalité globale du lupus par la réduction surtout des évènements thrombotiques. Au vu de tous ces éléments, une prise en charge globale des FRCV au cours du lupus est ainsi préconisée. En effet, selon les dernières recommandations du groupe de travail français du réseau des centres de référence et de compétence du lupus systémique, une statine doit être prescrite en prévention primaire, en fonction du taux de LDL cholestérol et du nombre de facteurs de risque cardiovasculaires en considérant le lupus comme un facteur de risque supplémentaire et en prévention secondaire lorsque le taux de LDL cholestérol dépasse 0,70 g/l [5]. Il est recommandé également de prescrire de l'aspirine en prévention primaire aux patients lupiques ayant un risque d’évènement cardiovasculaire fatal de 5% à 10 ans et ce en l'absence de contre-indications [5].

## Conclusion

Devant l'importance du risque cardiovasculaire au cours du LES démontré dans plusieurs études et confirmé par nos résultats, une surveillance rapprochée des FDRCV semble primordiale. Ainsi le LES doit être considéré comme un facteur de risque CV à part entière. Il serait souhaitable également de limiter la prescription des corticoïdes et d'assurer le contrôle du bilan lipidique par les statines
